# CBNA: A control theory based method for identifying coding and non-coding cancer drivers

**DOI:** 10.1371/journal.pcbi.1007538

**Published:** 2019-12-02

**Authors:** Vu V. H. Pham, Lin Liu, Cameron P. Bracken, Gregory J. Goodall, Qi Long, Jiuyong Li, Thuc D. Le

**Affiliations:** 1 School of Information Technology and Mathematical Sciences, University of South Australia, Mawson Lakes, Australia; 2 Centre for Cancer Biology, an alliance of SA Pathology and University of South Australia, Adelaide, Australia; 3 Department of Medicine, The University of Adelaide, Adelaide, Australia; 4 Perelman School of Medicine, University of Pennsylvania, Philadelphia, Pennsylvania, United States of America; Ottawa University, CANADA

## Abstract

A key task in cancer genomics research is to identify cancer driver genes. As these genes initialise and progress cancer, understanding them is critical in designing effective cancer interventions. Although there are several methods developed to discover cancer drivers, most of them only identify coding drivers. However, non-coding RNAs can regulate driver mutations to develop cancer. Hence, novel methods are required to reveal both coding and non-coding cancer drivers. In this paper, we develop a novel framework named Controllability based Biological Network Analysis (CBNA) to uncover coding and non-coding cancer drivers (i.e. miRNA cancer drivers). CBNA integrates different genomic data types, including gene expression, gene network, mutation data, and contains a two-stage process: (1) Building a network for a condition (e.g. cancer condition) and (2) Identifying drivers. The application of CBNA to the BRCA dataset demonstrates that it is more effective than the existing methods in detecting coding cancer drivers. In addition, CBNA also predicts 17 miRNA drivers for breast cancer. Some of these predicted miRNA drivers have been validated by literature and the rest can be good candidates for wet-lab validation. We further use CBNA to detect subtype-specific cancer drivers and several predicted drivers have been confirmed to be related to breast cancer subtypes. Another application of CBNA is to discover epithelial-mesenchymal transition (EMT) drivers. Of the predicted EMT drivers, 7 coding and 6 miRNA drivers are in the known EMT gene lists.

## Author summary

Cancer is a disease of cells in human body and it causes a high rate of deaths worldwide. There has been evidence that coding and non-coding RNAs are key players in the initialisation and progression of cancer. These coding and non-coding RNAs are considered as cancer drivers. To design better diagnostic and therapeutic plans for cancer patients, we need to know the roles of cancer drivers in cancer development as well as their regulatory mechanisms in the human body. In this study, we propose a novel framework to identify coding and non-coding cancer drivers (i.e. miRNA cancer drivers). The proposed framework is applied to the breast cancer dataset for identifying drivers of breast cancer. Comparing our method with existing methods in predicting coding cancer drivers, our method shows a better performance. Several miRNA cancer drivers predicted by our method have already been validated by literature. The predicted cancer drivers by our method could be a potential source for further wet-lab experiments to discover the causes of cancer. In addition, the proposed method can be used to detect drivers of cancer subtypes and drivers of the epithelial-mesenchymal transition in cancer.

## Introduction

**Fig 1:**
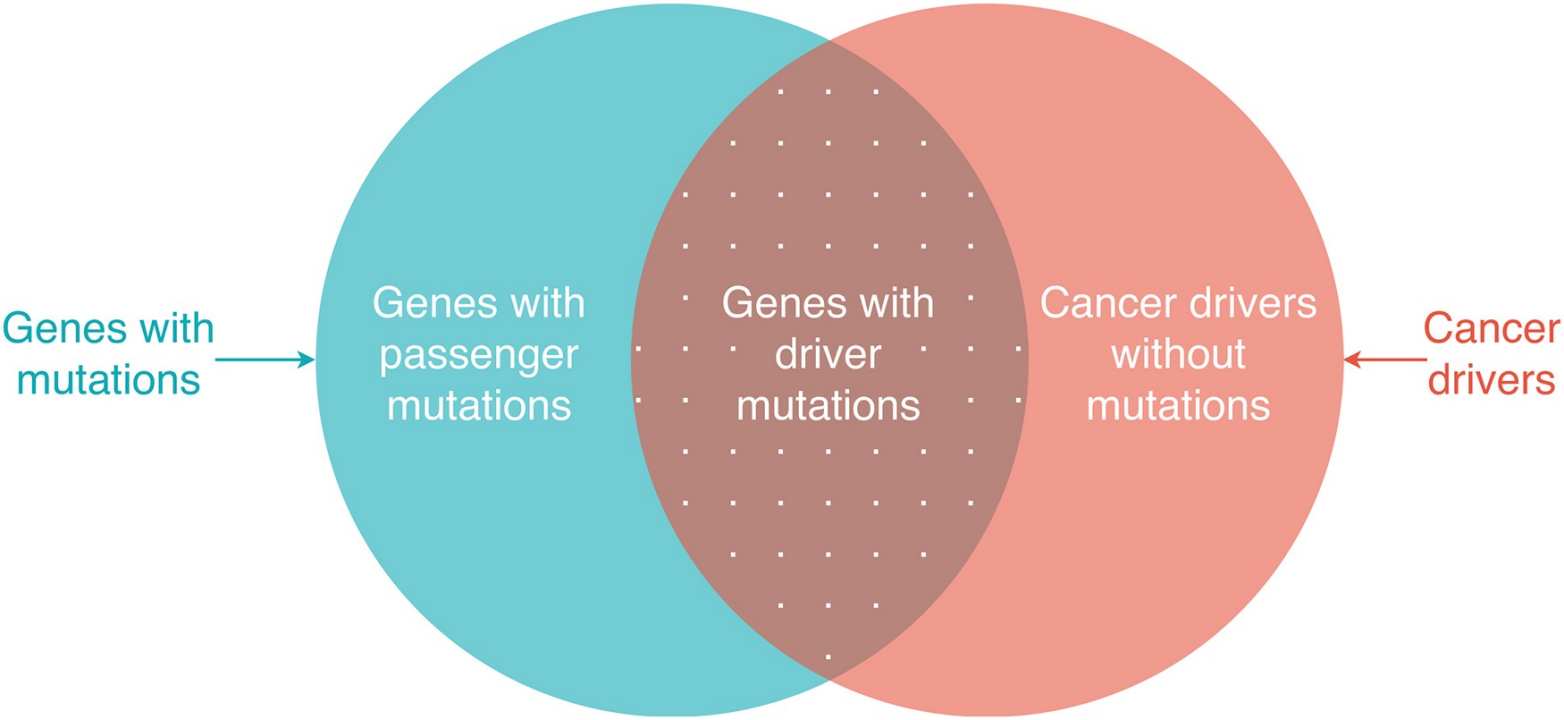
Coding cancer drivers and genes with mutations. Genes with driver mutations are cancer drivers. Some genes which do not bear mutations but regulate driver mutations to progress cancer are also considered as cancer drivers.

As cancer driver genes (cancer drivers for short) play significant roles in cancer development and progression, identifying cancer drivers and their regulatory mechanism is critical in the design of effective cancer treatments. There has been evidence that cancer drivers are related to gene mutations. Mutations in the genome can be single-nucleotide variants (SNVs), insertions and deletions (indels), copy number aberrations (CNAs), or structural variants (SVs) [1]. These mutations might cause normal cells to transform to tumour cells, resulting in cancer initialisation and development. For instance, it has been confirmed that the mutations in AKT1 and BRCA1 genes cause breast cancer [2] and the mutations in MET and VHL genes are related to kidney cancer [3]. Nevertheless, some mutations might not progress cancer. Mutations which have impacts on cancer development are driver mutations while mutations which do not play any role in cancer development are passenger mutations [4, 5]. Genes that bear driver mutations are considered as cancer drivers [6]. However, some genes, which do not bear mutations but do regulate driver mutations to progress cancer, are also considered as cancer drivers as shown in Fig 1. Moreover, cancer drivers can also be non-coding RNAs since non-coding regions account for around ninety eight percent of the human genome [7] and non-coding RNAs are proved to be related to cancer development [8, 9].

A wide range of computational methods utilising various types of genomic data have been developed to identify cancer drivers and their regulatory mechanisms behind the cancer initialisation and progression. In general, these computational methods can be categorised into two main approaches: mutation-based approach and network-based approach. Mutation-based approach includes methods which classify driver mutations and passenger mutations mainly based on mutations and their characteristics, i.e. functional impact [10], recurrence [11–14], enrichment in externally defined regions [15], mutual exclusivity [4, 16], etc. Particularly, OncodriveFM [10] evaluates the functional impacts of gene mutations to discover cancer drivers based on the hypothesis that genes which have a variation with significantly functional impacts can be candidate driver genes. OncodriveCLUST [11] hypothesises that gain-of-function mutations largely cluster in particular protein sections and the proposed method reveals cancer driver genes whose mutation clustering is largely biased. ActiveDriver [15] discovers driver genes which are enriched in mutations located in post-translationally modified sites. WeSME [16] and CoMEt [4] identify cancer driver genes by utilising statistical tests to evaluate the mutual exclusivity of genomic events and candidate cancer drivers are genes which have mutations with a significantly mutual exclusivity. One more example in the mutation-based approach is CHASM [17], which applies random forest, a machine learning technique, to detect driver mutations.

The second main approach includes network-based methods which identify cancer drivers by evaluating the role of genes in a biological network [18–24]. A typical method in this category is DawnRank [18], a ranking framework that applies PageRank [25, 26] to assess the impact of genes in a gene interaction network. DriverNet [19] integrates an influence graph, genome data, and transcriptome data to detect driver genes. Like WeSME [16] and CoMEt [4], MEMo [20] also relies on the mutual exclusivity of mutations but the method combines the mutation information with network information to identify mutual exclusivity modules in networks. TieDIE [21] applies network diffusion to detect cancer drivers based on the relationship of genomic events and changes in cancer subtypes. iMCMC [22] uses network information to identify mutated core modules in cancer.

The two types of methods have their own advantages and limitations. Mutation-based methods are easy to be applied to different mutation datasets as they are mainly based on mutation data. However, their applications are limited due to the incompleteness of mutation databases. Network-based methods are able to elucidate molecular mechanisms in developing diseases at the network level [27, 28], but they usually require large datasets to generate reliable results. Furthermore, most current methods use general networks which are not specific to any cancer type. Thus, these networks might include some interactions which do not exist in a certain cancer type. Another potential limitation of network-based methods such as DawnRank and DriverNet is that they discover only candidate drivers which alter other genes’ expression. However, some drivers may not change other genes’ expression, or some genes accidentally alter other genes’ expression although they are not drivers. In addition, current methods detect coding drivers though cancer drivers can also be non-coding RNAs. Therefore, there is a strong need for effective methods to find both coding and non-coding drivers and their regulatory relationships that drive cancers.

With the aim to detect both coding and non-coding drivers, in particular microRNA (miRNA) drivers, we develop a novel and effective method called Controllability based Biological Network Analysis (CBNA). We firstly build the network for a condition (e.g. cancer state) from the expression data of miRNAs, Transcription Factors (TFs), and mRNAs of cancer patients. We then combine this network with the protein-protein interaction (PPI) network [29] and filter out edges of the network learned from the expression data, which are not in existing databases, including miRTarBase [30], TarBase [31], miRWalk [32], TargetScan [33], and TransmiR [34]. Besides integrating miRNAs into the network, we utilise the gene expression of patient cohort in building it. Thus, we eliminate interactions which do not exist in a particular cancer type and the resulting network is specific to that cancer type. We will then discover drivers based on the network.

To overcome the limitation of the current methods which are based on the effect of potential drivers on downstream genes’ expression in the network as the above discussion, we might need to have a more effective method to evaluate the role of genes in the network. Inspired by control theory [35] and its application in detecting a subset of nodes in a network which can control the whole network [36], we apply them to analyse the above network. Control theory has a wide range of applications, from electric circuits or manufacturing processes to spacecraft or robots. According to control theory, a system is controllable if we can drive it from any state to any expected state in a time frame by suitable inputs. Based on control theory, the idea of network control was introduced in [36] to capture the state of how a subset of nodes in a network, known as critical nodes, control the whole network at a time. Following this idea, we apply the method in [36] to identify the critical nodes in the network learned above. We consider the critical nodes as the driver genes in the network as they play the central roles in the network of cancer state and likely control that cancer condition. Finally, we use the mutation data (i.e. somatic mutations) to compute mutation frequency of genes to rank the predicted cancer drivers.

We apply the proposed method to the breast invasive carcinoma (BRCA) dataset of The Cancer Genome Atlas (TCGA) [37] to identify breast cancer drivers. The predicted breast cancer drivers include coding drivers with mutations, coding drivers without mutations, and miRNA drivers. We validate the coding drivers with mutations using Cancer Gene Census (CGC) [38] and the result shows that the proposed method outperforms the existing methods, including OncodriveFM [10], OncodriveCLUST [11], ActiveDriver [15], DawnRank [18], DriverNet [19], and NetSig [39]. Several predicted coding drivers without mutations are enriched in molecular functions and biological processes, suggesting their important roles in the human body and the effectiveness of the method. Moreover, we discover 17 miRNA drivers for breast cancer, some of which have been validated by literature and the rest can be good candidates for wet-lab validation.

We go further to study subtype-specific drivers by comparing the controllability of networks of different cancer subtypes. We predict several drivers which are specific to breast cancer subtypes as well as some genes which drive more than one subtype. In addition, we also apply CBNA to detect drivers of epithelial-mesenchymal transition (EMT) [40] and some discovered drivers are mesenchymal genes [41] or pro-mesenchymal miRNAs [42]. These results demostrate that CBNA is useful not only in identifying cancer drivers but also in predicting drivers for other processes such as EMT. Thus, CBNA provides a promising framework to study molecular mechanisms of the development of cancer and other diseases.

## Materials and methods

### Materials

In this project, we apply the proposed method CBNA to the BRCA dataset of TCGA [37]. This dataset contains the expression data of miRNAs, TFs, and mRNAs of 747 samples. The TF list, which is used to find which genes are TF genes in the expression dataset, is obtained from the work of [43]. CBNA also employs the directed PPI network of [29]. Besides, the method uses various databases of miRNA-TF and miRNA-mRNA interactions, including miRTarBase version 6.1 [30], TarBase version 7.0 [31], miRWalk version 2.0 [32], TargetScan version 7.0 [33], and a database for TF-miRNA interactions, TransmiR version 2.0 [34]. The mutation data in this study is also acquired from TCGA. All the datasets used in this paper are available at https://github.com/pvvhoang/CancerDriver.

### Controllability based Biological Network Analysis (CBNA)

#### Overview

**Fig 2:**
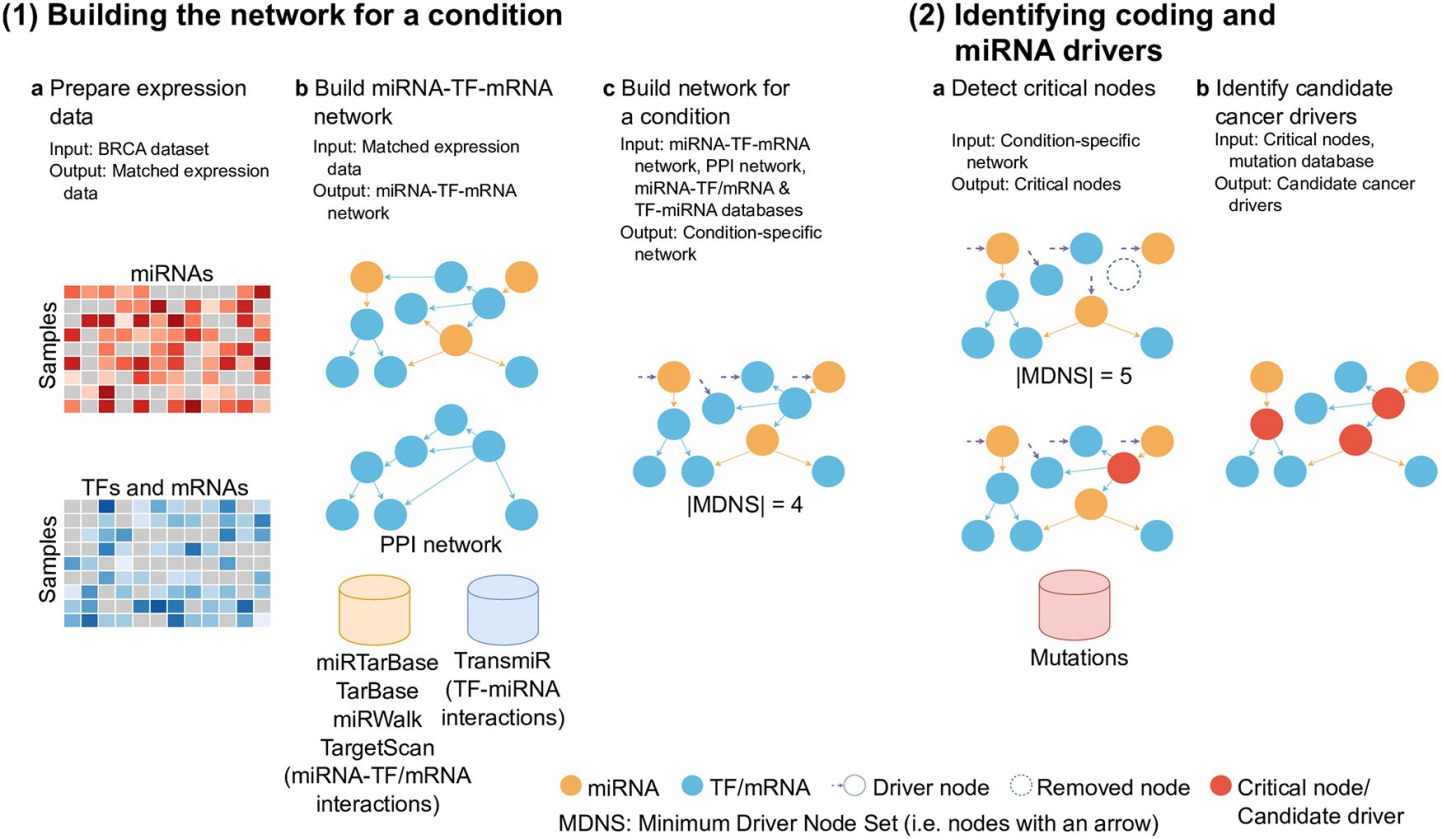
An illustration of CBNA. (1) Building the network for a condition: (a) Prepare matched miRNA and TF/mRNA expression data, (b) Build miRNA-TF-mRNA network where nodes represent miRNAs/TFs/mRNAs and an edge between two nodes indicates there is a significant Pearson correlation between the expression of the two nodes, (c) Create the network by combining the miRNA-TF-mRNA network with the PPI network and other existing databases, and (2) Identifying coding and miRNA drivers: (a) Detect critical nodes, (b) Identify candidate cancer drivers.

As shown in Fig 2, CBNA has two stages: (1) Building the network for a condition, and (2) Identifying coding and miRNA drivers. The detail of CBNA is described in the following sections.

#### Identifying cancer drivers with controllability analysis

**Fig 3:**
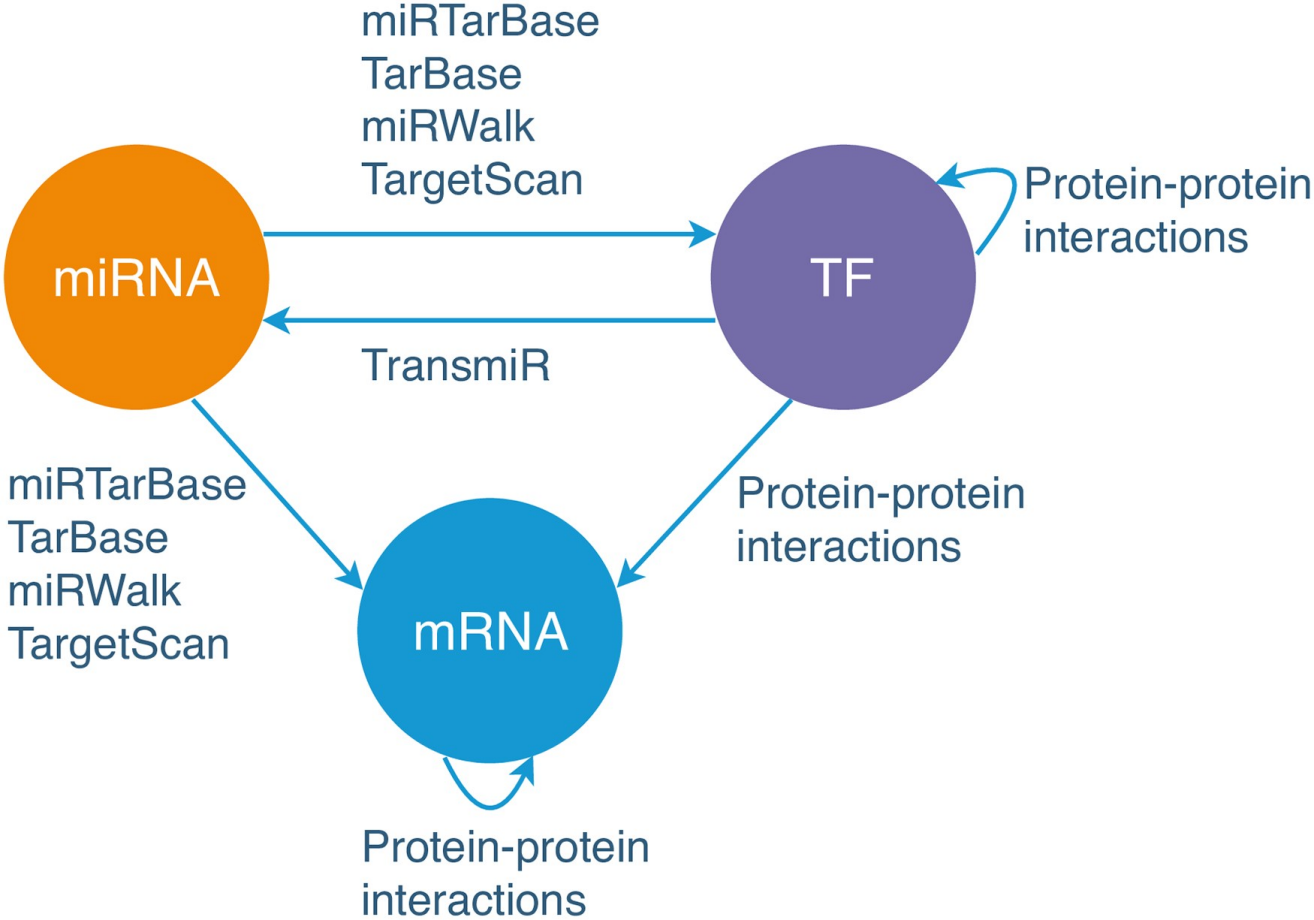
Determining the directions of edges in the miRNA-TF-mRNA regulatory network. In a miRNA-TF-mRNA regulatory network, miRNAs can regulate TFs and mRNAs, TFs can regulate miRNAs and mRNAs, TFs/mRNAs can regulate other TFs/mRNAs. This motif is adapted from the work of [66]. In addition, the databases used to filter out edges of the network are shown on arrows.

1. **Building the network for a condition**
  At the first stage, CBNA builds the network for a condition in three steps as described in the following.
  - Step 1a: Prepare the expression data of miRNAs, TFs, and mRNAs. We extract the expression data of matched samples of miRNAs and coding genes from the BRCA dataset [37]. In total, 747 samples are obtained. As the number of coding genes are over twenty thousand, we only select coding genes which are in the PPI network [29]. We then use the TF list in [43] to categorise the coding genes into two subsets, TFs and mRNAs. Finally, we have the expression data of 1,719 miRNAs, 839 TFs, and 5,168 mRNAs.
  - Step 1b: Build the miRNA-TF-mRNA network. We build the miRNA-TF-mRNA network for cancer state based on the above expression data. We firstly identify all pairwise Pearson [44] correlation coefficients (PCC) of all the nodes. We then calculate the significance of PCCs and apply an FDR cutoff (i.e. 0.05) to retain edges whose adjusted p-value is less than 0.05. The directions of the edges are determined as shown in Fig 3. In particular, miRNAs can regulate TFs & mRNAs, TFs can regulate miRNAs & mRNAs, and TFs/mRNAs can regulate other TFs/mRNAs.
  - Step 1c: Create the condition-specific network. To retain the ‘true’ interactions of coding genes, we firstly update the miRNA-TF-mRNA network with the PPI network by removing the edges between coding genes which are not in the PPI network. The PPI network is selected as it is a directed network, thus it can be used to combine with the directed miRNA-TF-mRNA network built in Step 1b. We then refine the obtained network by removing the edges if they are not in databases miRTarBase, TarBase, miRWalk, TargetScan, or TransmiR. As the network is obtained based on both expression data and existing databases, it is more reliable and specific to a certain cancer type. The final cancer condition-specific network consists of 7,726 nodes (including 1,719 miRNAs, 839 TFs, and 5,168 mRNAs) and 128,264 edges (inlcuding 16,087 miRNA-TF edges, 73,347 miRNA-mRNA edges, 18,950 TF-miRNA edges, 1,812 TF-TF edges, 1,188 TF-mRNA edges, and 16,880 mRNA-mRNA edges).
  As the motif shown in Fig 3, TF-TF/mRNA and mRNA-mRNA interactions of the miRNA-TF-mRNA network from Step 1b are refined with the PPI network. miRNA-TF/mRNA interactions are refined with miRTarBase, TarBase, miRWalk, TargetScan and TF-miRNA interactions are refined with TransmiR.
2. **Identifying coding and miRNA drivers**
  At the second stage, CBNA identifies drivers from the built network with the following two steps.
  - Step 2a: Detect critical nodes of the built network. According to the network control idea [36] (Details are introduced in the next section), a network can be fully controlled by a minimum set of nodes called minimum driver node set (MDNS). Applying this network control idea, we detect the MDNS of the network obtained from stage 1. Then we discover critical nodes of the network. The critical nodes are nodes whose absence causes a rise in the size of the MDNS. It means that when these critical nodes are removed from the network, more interactions on nodes (i.e. interactions on driver nodes) are needed to control the whole network.
  - Step 2b: Identify candidate cancer drivers. As without the critical nodes, we need to interact on more driver nodes to control the whole regulatory network, the critical nodes play the central role in controlling the whole network and alterations in these nodes such as over expression or mutation might transform the state of a person from normal to cancer. Thus, these critical nodes in the network obtained from Stage 1 can be considered as candidate cancer drivers. We categorise the candidate cancer drivers into three subsets, coding drivers with mutations, coding drivers without mutations, and miRNA drivers. As most of the predicted drivers are coding drivers with mutations, we rank predicted coding drivers with mutations to get significant candidates. We download the mutation data of the BRCA samples from TCGA. Based on the variant classification of mutations, we only select mutations which are functional, such as splice_site, in_frame_del, frame_shift_del, etc. and compute the mutation frequency of coding genes. The more frequent the mutation of a coding driver is, the higher it is in the ranking list.

#### Controllability of complex networks

The idea of the network control [36] is that a directed network can be guided by a subset of nodes known as driver nodes. The mathematical theory behind this network control idea is described as below.

Suppose that we have a system with *N* nodes *x*_1_, …, *x*_*N*_. The following *N*x*N* matrix *A* captures the interaction strength between nodes:

$$\begin{array}{c} A=(\begin{array}{cccc} a_{11} & a_{12} & \cdots & a_{1N}\\ a_{21} & a_{22} & \cdots & a_{2N}\\ \vdots & \vdots & \ddots & \vdots \\ a_{N1} & a_{N2} & \cdots & a_{NN} \end{array}), \end{array}$$ (1)

where *a*_*ij*_ represents the edge strength of node *j* on node *i* (*i*, *j* ∈ {1, …, *N*}). If there is no edge from node *j* to node *i* then *a*_*ij*_ = 0.

Let *B*_*N*×*M*_ be the input matrix (*M* ≤ *N*) which indicates *M* nodes controlled by an external controller:

$$\begin{array}{c} B=(\begin{array}{cccc} b_{1} & 0 & \cdots & 0\\ 0 & b_{2} & \cdots & 0\\ \vdots & \vdots & \ddots & \vdots \\ 0 & 0 & \cdots & b_{M}\\ \vdots & \vdots & \ddots & \vdots \\ 0 & 0 & \cdots & 0 \end{array}), \end{array}$$ (2)

where *b*_*i*_ represents the interaction strength of the controller on node *i* (*i* ∈ {1, …, *M*}).

Based on Kalman’s controllability condition [35], the network represented by the matrix *A* is controllable through the *M* nodes indicated in *B* (these *M* nodes are called driver nodes) if and only if the controllability matrix *C*_*N*×*NM*_ satisfies the following:

$$\begin{array}{c} rank(C)=N, \end{array}$$ (3)

where *C* is a combination of matrices *B*, *AB*, *A*^2^*B*, …, *A*^*N*−1^*B* and represented as *C* = (*B*, *AB*, *A*^2^*B*, …, *A*^*N*−1^*B*).

Intuitively, the rank of the controllability matrix *C* being *N* indicates that all *N* variables (i.e. *N* nodes of the network) are controllable. In addition, it can be noted that as we just need to identify the rank of *C*, we do not need to compute the value of *C*. The condition shown in Eq (3) can be satisfied if it is possible to select non-zero link weights in *A* and *B*. Therefore, this method can also be applied to networks without the weight of links among nodes.

We may identify several sets of nodes which can satisfy the condition Eq (3). However, we are interested in discovering the minimum number of driver nodes (i.e. minimum *M*), called minimum driver node set (MDNS), whose control is sufficient to control the whole network. In step 2a of the second stage of CBNA, applying this method, we identify the MDNS of the miRNA-TF-mRNA network. Then we detect critical nodes for our network by removing node by node out of the network, if the absence of a node increases the size of the MDNS, it is a critical node.

Using the network control, our method can discover driver genes which are coding RNAs without mutations or miRNAs, which are missed by other cancer driver identification methods. In addition, since our method allows to build the network based on the expression data, it can be applied to detect drivers for any condition or disease other than cancer drivers.

#### Implementation

The proposed framework has been developed in R and its source code as well as the scripts for reproducing the experiment results in this study are available at https://github.com/pvvhoang/CancerDriver.

## Results and discussion

### Characterising the controllability of the miRNA-TF-mRNA network

**Fig 4:**
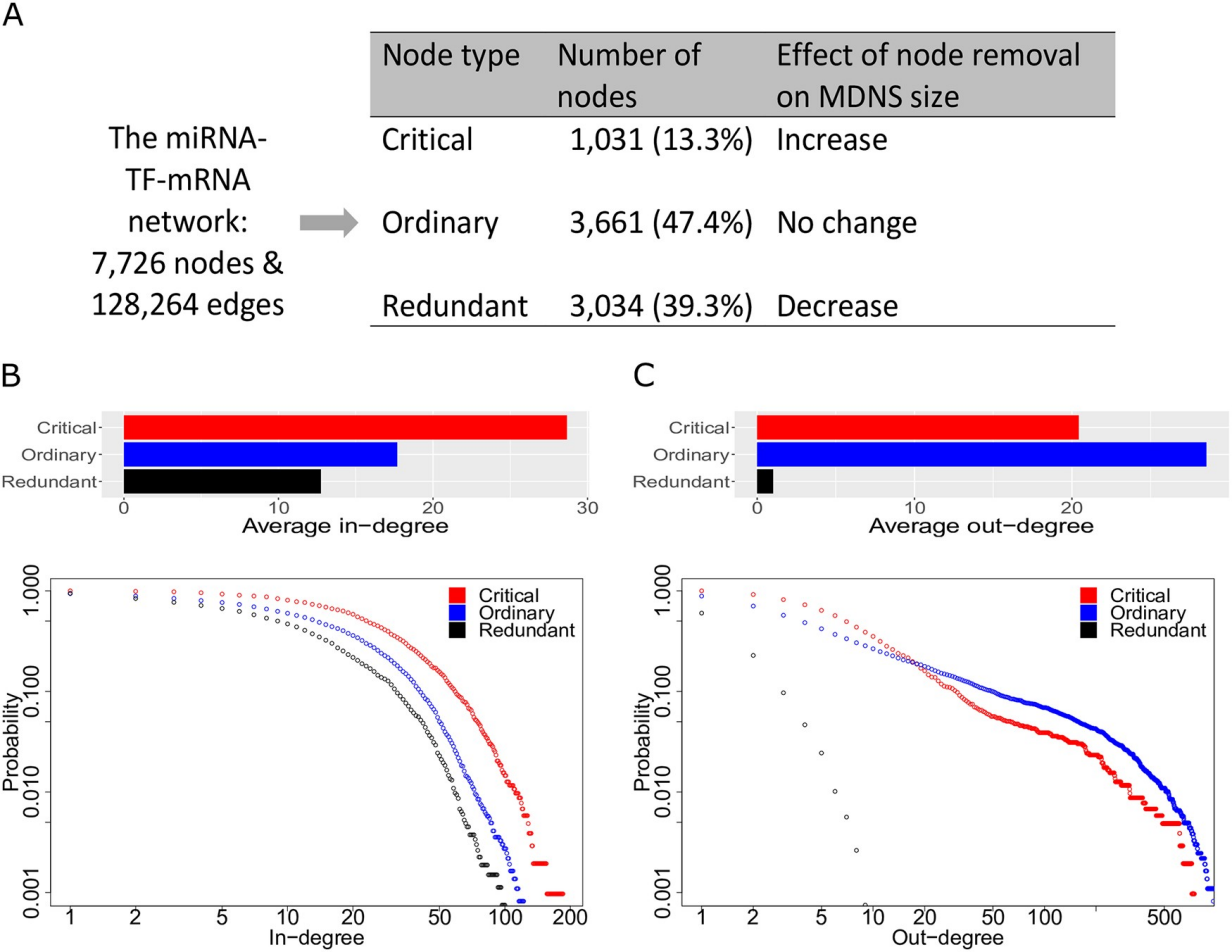
Characterising the controllability of the miRNA-TF-mRNA network. (A) Identification of critical, ordinary, and redundant nodes in the network. (B) Average in-degree and *accumulative* in-degree distribution (i.e. the in-degree i with the probability p means that the probability to pick a node which has in-degree larger than or equal to i is p) for three different node types. (C) Average out-degree and *accumulative* out-degree distribution for three different node types.

The miRNA-TF-mRNA network obtained by CBNA consists of 7,726 nodes and 128,264 directed edges. We apply the method in [36] to evaluate the controllability of the network by identifying its MDNS. Although the MDNS is not unique, all the MDNS sets identified in the BRCA network are of the same size (i.e. contain the same number of nodes, denoted as *N*_*D*_). The identified MDNS contains 2,877 nodes (i.e. *N*_*D*_ is 2,877), accounting for 39.1% of the nodes in the constructed miRNA-TF-mRNA network. We then classify the nodes in the miRNA-TF-mRNA network as critical, ordinary, and redundant based on the change of *N*_*D*_ upon their removal. A node is critical if its removal increases *N*_*D*_, ordinary if removing it does not change *N*_*D*_, and redundant if removing it decreases *N*_*D*_. In the miRNA-TF-mRNA network, 13.3% of nodes are critical, 47.4% are ordinary, and the remaining 39.3% are redundant (Fig 4A). We find that critical nodes have higher in-degrees compared with ordinary and redundant nodes, which can be seen in the average in-degree and accumulative in-degree distributions of nodes in Fig 4B. From Fig 4C, the out-degrees of critical nodes are high, although ordinary nodes have higher out-degrees.

### CBNA is effective in detecting coding cancer drivers with mutations

In this section, we compare the performance of the proposed method CBNA with six existing methods for identifying cancer drivers, OncodriveCLUST [11], ActiveDriver [15], OncodriveFM [10], DriverNet [19], DawnRank [18], and NetSig [39]. As these methods are developed to discover coding cancer drivers, we only compare these six methods with CBNA in discovering mutated coding cancer drivers. These methods are selected as they are representatives of different approaches for uncovering cancer drivers. OncodriveCLUST, ActiveDriver, and OncodriveFM are mutation-based methods while DriverNet, DawnRank, and NetSig are network-based methods. OncodriveCLUST deals with the clustering of mutations in genes, ActiveDriver detects enrichment of mutated genes in externally defined regions, and OncodriveFM evaluates the functional impacts of mutations. For the three network-based methods, DriverNet combines an influence graph with genome data and transcriptome data to discover driver genes, DawnRank applies PageRank to rank the impact of genes in the network, and NetSig integrates protein interaction networks and tumour data to predict driver genes. Since these methods have different assumptions for identifying cancer drivers, each method may miss some particular driver genes. For example, OncodriveCLUST would miss tumour genes with broad mutation patterns as it is based on the clustering of mutations and DawnRank would miss some drivers which do not alter the expression of other genes as it uses the impact of genes.

For the experiments in this paper, we use the BRCA dataset of TCGA [37], including the expression data and the mutation data. For the interaction network required by DriverNet, we use the network from the paper [45]. The network used by DawnRank in our experiments is obtained directly from the authors of the method [18]. The network used by our proposed method CBNA is described in Section Materials and methods.

We utilise the Cancer Gene Census (CGC) from the COSMIC database [46] as a groundtruth for coding driver genes. CGC is a commonly used cancer gene database in cancer research for validating cancer drivers predicted by computational methods. We measure performance of a method based on the number of uncovered cancer driver genes which are in CGC. The higher the number of validated cancer driver genes a method has discovered, the better the method is.

To facilitate the comparison, the top cancer driver genes (top 50, 100, 150, and 200 respectively) predicted by each of the seven methods are chosen to be validated with the CGC. OncodriveCLUST, OncodriveFM, and NetSig order their discovered cancer driver genes based on a corrected p-value. The results of ActiveDriver and DriverNet are ordered by p-value. The cancer drivers predicted by DawnRank are sorted by the ranking scores used by the method, and the ranking of identified cancer drivers by CBNA is based on the mutation frequency of genes.

**Fig 5:**
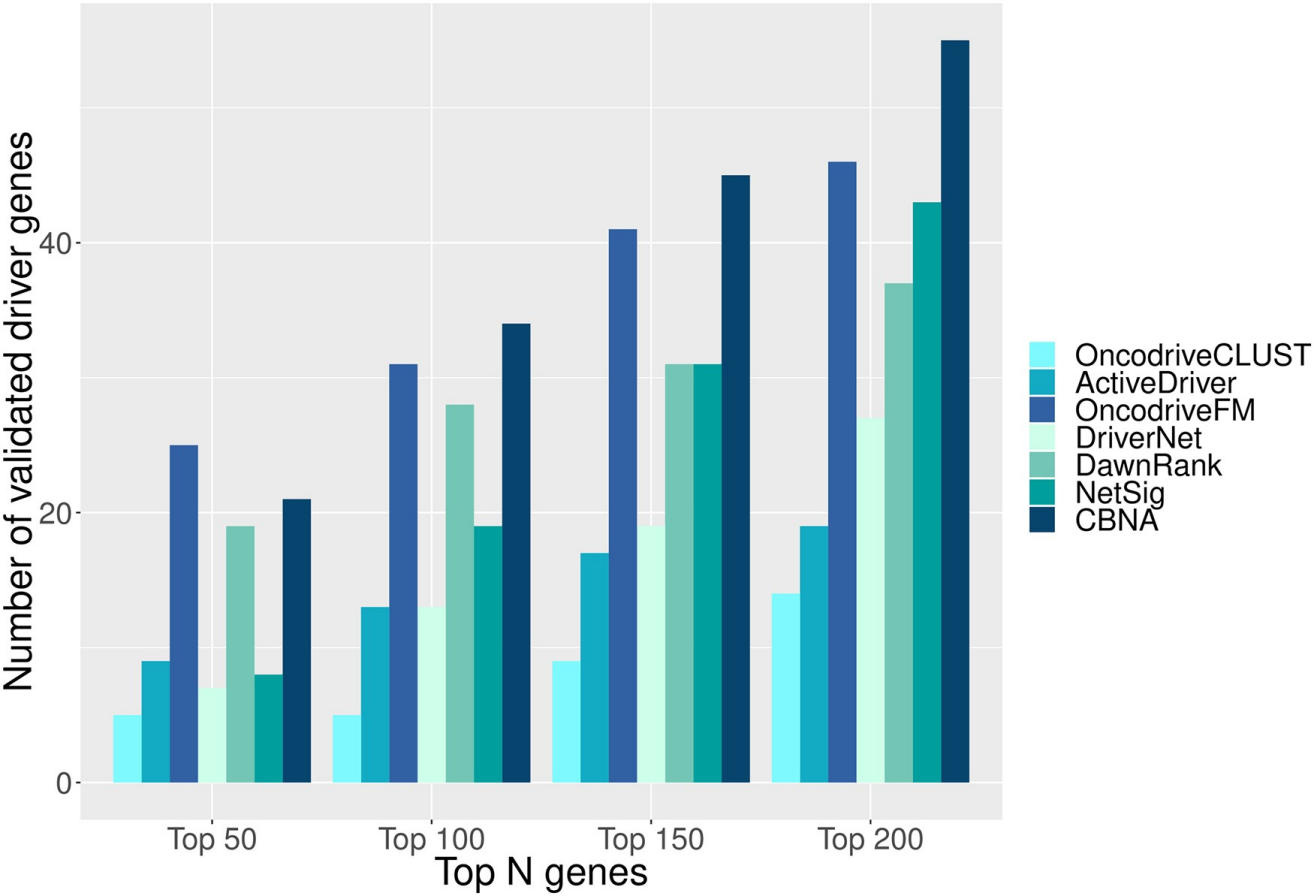
Validation using CGC. The cancer drivers predicted by each method are validated against CGC. Each bar in the chart indicates the number of validated coding driver genes for each method.

The result of the comparison is shown in Fig 5. In the case of the top 50 cancer drivers predicted by the methods, the CBNA is comparable to OncodriveFM and they outperform the other five methods. In the cases of top 100, 150, and 200 discovered driver genes, CBNA outperforms the other given methods. In addition, instead of ranking predicted coding cancer drivers based on the mutation frequency of genes, we also rank predicted genes based on mutation frequency and spectrum of patients, mutation rates of genes incorporating expression level and replication time, and functional impact of mutations (see the detail in Supplementary section 1 in S1 Text).

To have a more comprehensive comparison, we also use the following three measures, *Precision*, *Recall*, and *F*_1_ *Score* computed based on CGC and the top N genes (N is from 1 to 200) predicted by the seven methods:

$$\begin{array}{c} Precision=\frac{tp}{tp+fp}, \end{array}$$ (4)

$$\begin{array}{c} Recall=\frac{tp}{tp+fn}, \end{array}$$ (5)

$$\begin{array}{c} F_{1}Score=2*\frac{Precision*Recall}{Precision+Recall}, \end{array}$$ (6)

where *tp* represents the number of discovered cancer drivers which are in CGC, *fp* is the number of discovered cancer drivers which are not in CGC, and *fn* is the number of drivers which are in CGC but not discovered by the method.

**Fig 6:**
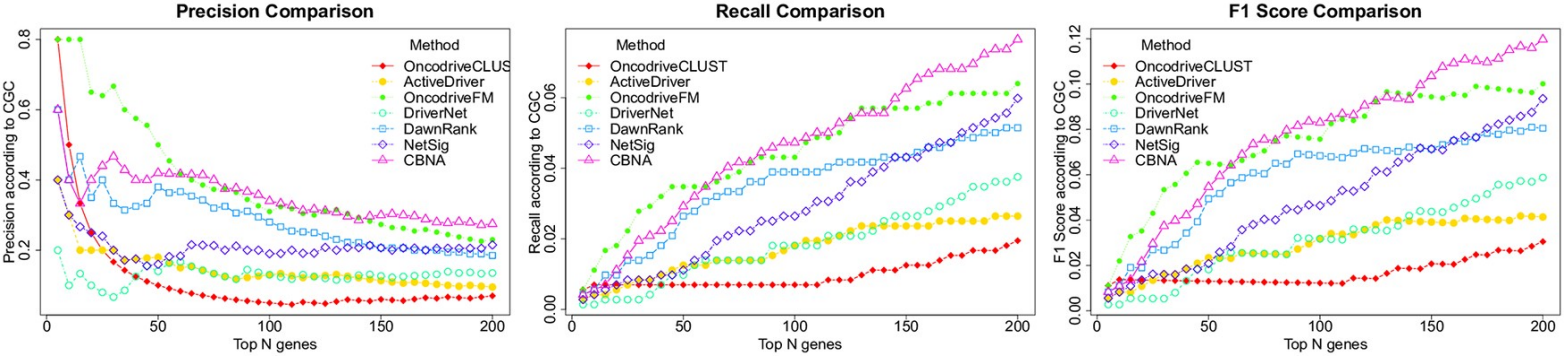
Comparison of Precision, Recall, and F_1_Score for the top ranking genes predicted by OncodriveCLUST, ActiveDriver, OncodriveFM, DriverNet, DawnRank, NetSig, and CBNA. In each diagram, the x-axis is the number of the top ranking genes. The y-axis is the value of *Precision*, *Recall*, or *F*_1_ *Score*.

The comparison result is shown in Fig 6. We see that although OncodriveFM has better or similar performance as CBNA when N is small, our method outperforms OncodriveFM and the other five methods when N becomes larger.

**Fig 7:**
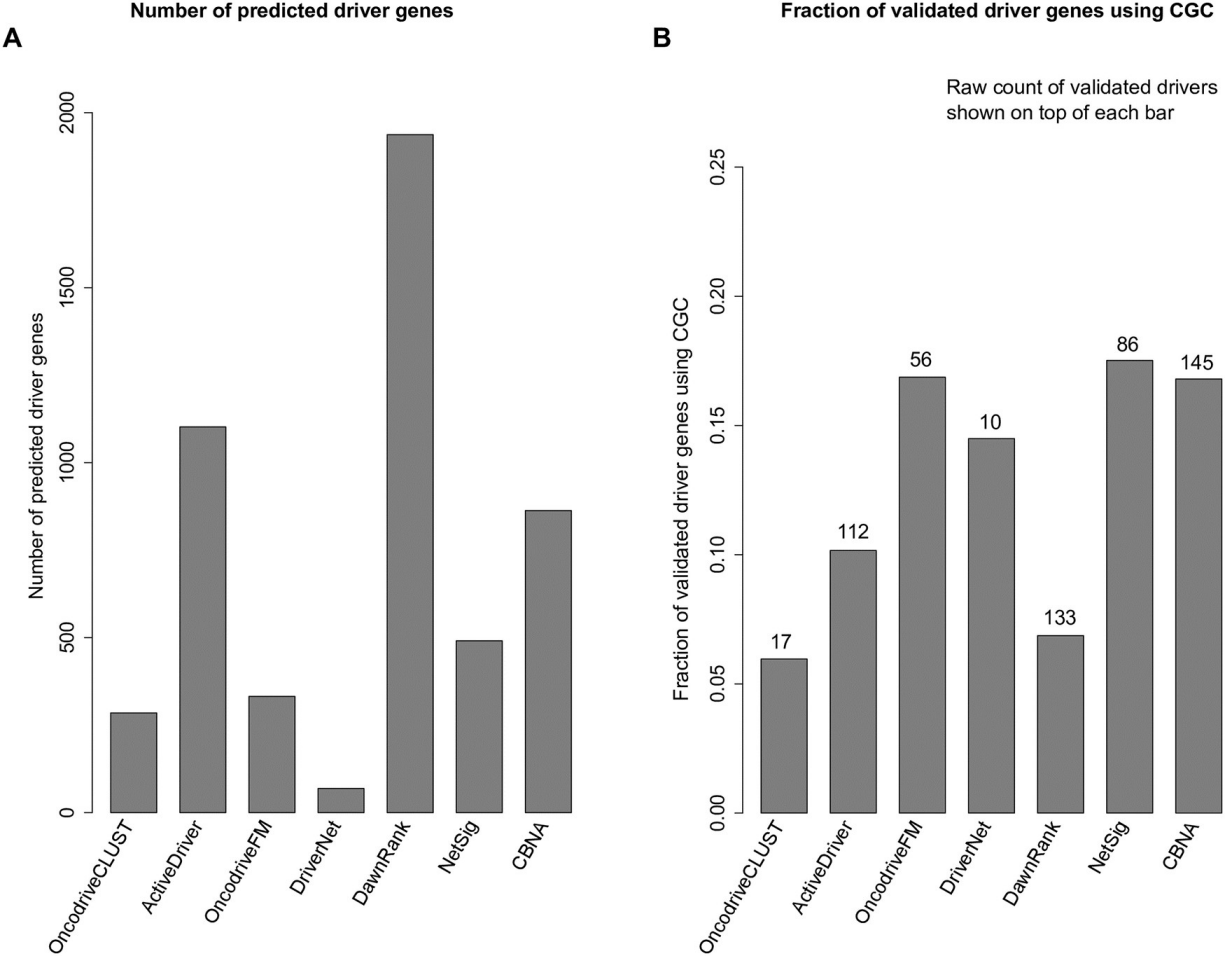
Evaluation based on the total number of predicted driver genes. (A) Number of predicted drivers, (B) Fraction of validated drivers in the CGC and raw count of predicted drivers indicated on top of each bar.

In the above evaluation, we looked at the top cancer driver genes predicted by the methods, now we evaluate the methods based on the *total* number of driver genes predicted by the methods, in the same way as the evaluation done by the study in [6]. The detailed result is shown in Fig 7, where we see that the total numbers of predicted drivers vary (*q* ≤ 0.1 for OncodriveCLUST and OncodriveFM (q-value is the corrected p-value), *p* ≤ 0.05 for ActiveDriver and DriverNet, adjusted p ≤0.05 for NetSig, all predicted genes by DawnRank and CBNA). ActiveDriver, DawnRank, NetSig, and CBNA predict around or over 500 driver genes, whereas the remaining has less than 500 genes. For each method, using CGC, we assess the fraction of validated driver genes among all the driver genes predicted by the method, and as shown in Fig 7B CBNA has the highest number of validated driver genes comparing to the other six methods.

Although CBNA outperforms other current benchmark methods in identifying cancer drivers, computational methods may never completely replace wet-lab experiments in validating the biological findings. However, the novel cancer drivers predicted by CBNA can be good candidates for further wet-lab experiments to confirm their roles in cancer initialisation and progression. As we construct the network using PCCs, the expression levels of a gene may not matter but the correlation of the expression levels of two genes matters. In saying so, including many lowly-expressed genes may cause false positives, and thus we provide an option pre-processing function to filter out those genes before applying CBNA to the dataset to identify cancer drivers.

**Fig 8:**
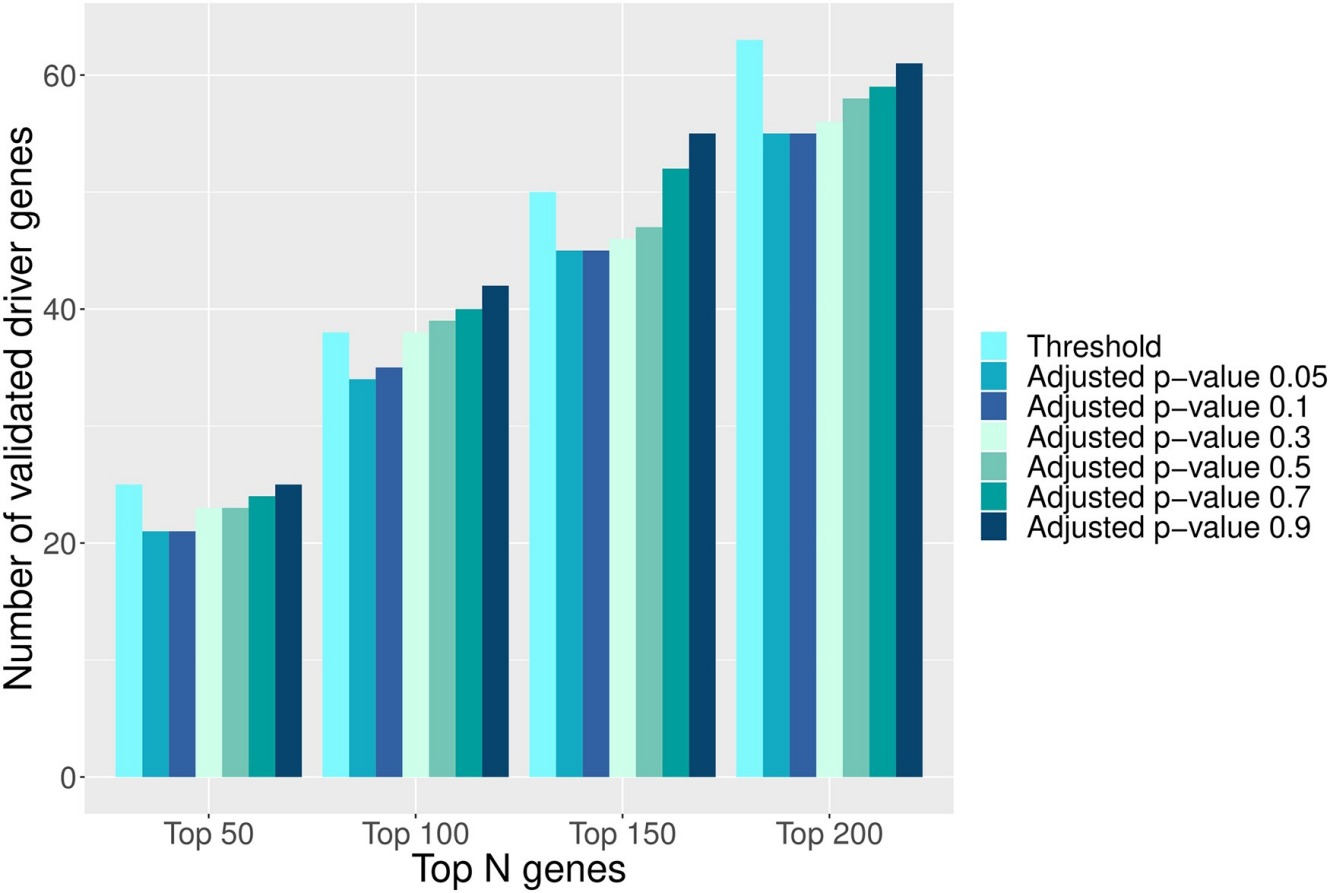
CBNA using different adjusted p-value cutoffs. The cancer drivers predicted by CBNA with different adjusted p-value cutoffs are validated by the CGC. Each bar in the figure shows the number of validated coding cancer drivers of CBNA with a cutoff.

To evaluate the impact of the adjusted p-value cutoff on the performance of CBNA, we run CBNA with different cutoff values and the results are shown in Fig 8. It can be seen that the performance of CBNA is quite consistent in different settings. It does not change much when we change the adjusted p-value thresholds.

**Fig 9:**
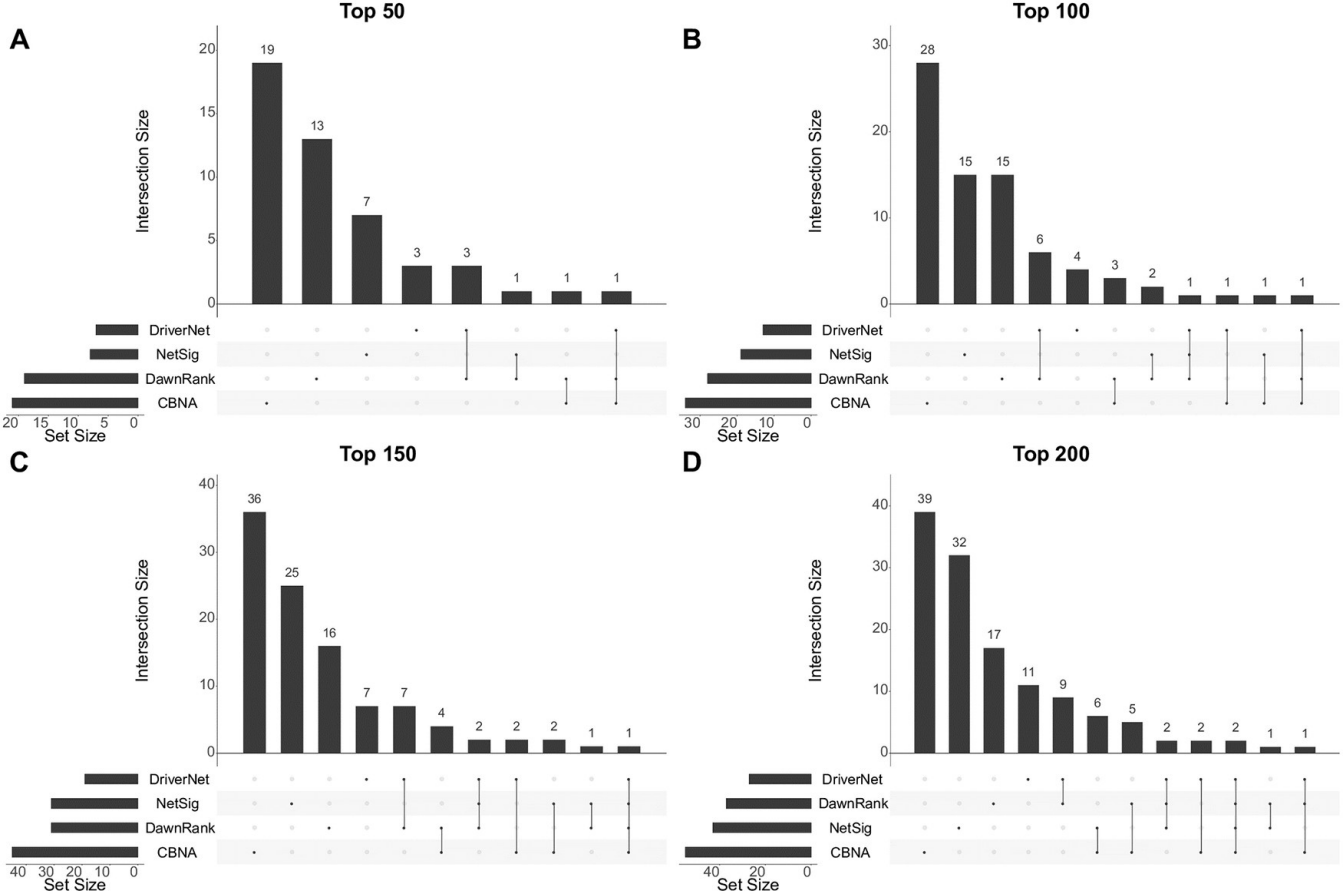
Overlap between different methods. The diagram shows the overlap among the four methods in their top 50, 100, 150, and 200 predicted drivers. For each of the four cases, the horizontal bars at the bottom left show the numbers of predicted cancer drivers validated by the CGC for the four methods; the vertical bars and the dotted lines together indicate the numbers of validated cancer drivers which overlap with each other.

In addition, to check if the selected network-based methods detect similar cancer drivers, we compare their results and the findings of the four methods have little overlap as indicated in Fig 9. In the figure, the top 50, 100, 150, and 200 cancer driver genes identified by these methods and validated against the CGC are intersected. Although there are some known cancer drivers uncovered by multiple methods, CBNA discovers some important known cancer drivers which are not identified by others. Since the results of these methods are complementary, they could be used together to maximize the effectiveness in predicting cancer drivers. Moreover, besides the known cancer drivers in the CGC, CBNA can detect novel cancer drivers which can be used as candidates in wet-lab experiments to confirm their roles in the cancer initialisation and progression.

**Fig 10:**
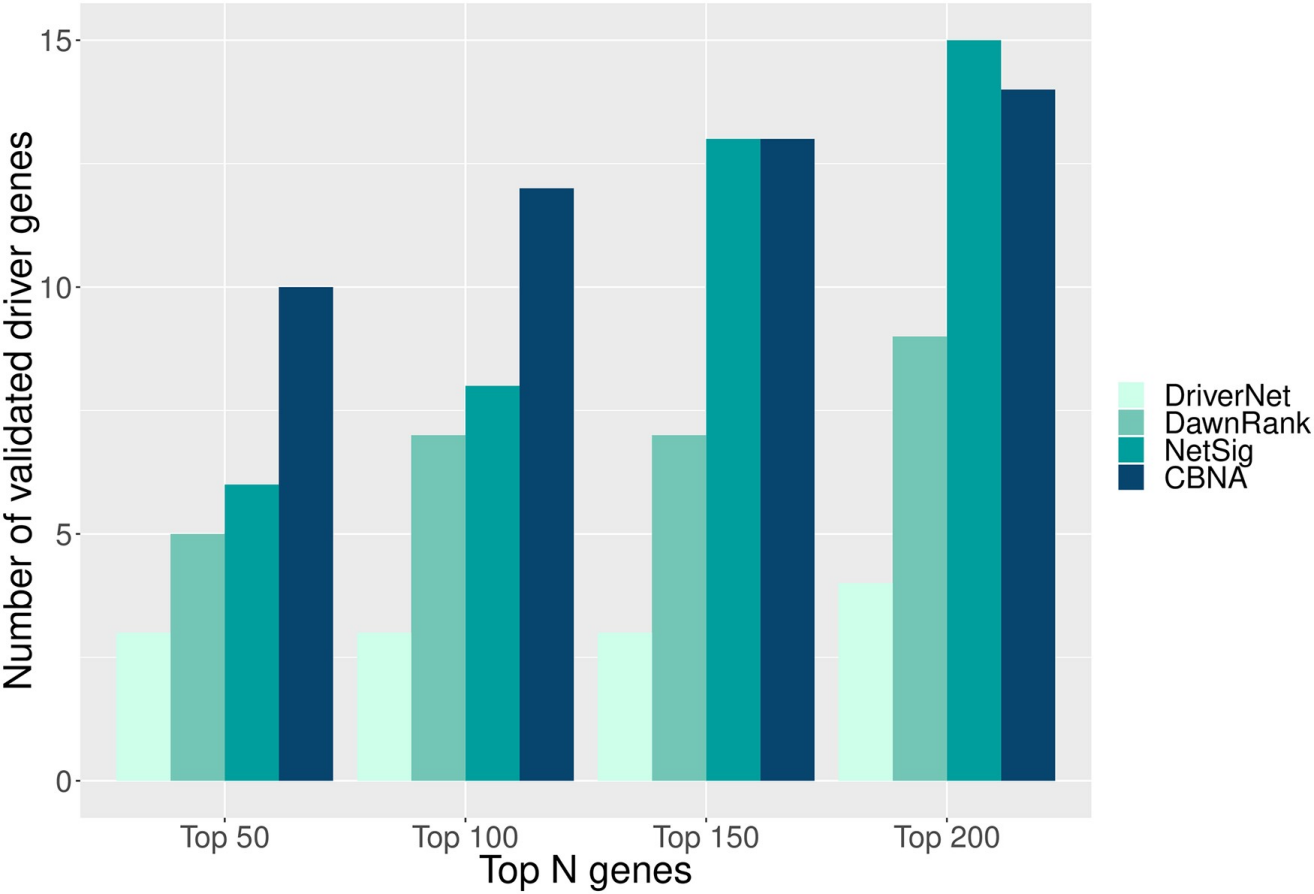
Validation using a well-curated set of breast cancer drivers. The cancer drivers predicted by the methods are validated by a well-curated set of breast cancer drivers. Each bar in the figure shows the number of validated coding cancer drivers of each method.

To further evaluate the performance of network-based methods in detecting breast cancer drivers, we validate the coding cancer drivers by these methods against a well-curated set of breast cancer drivers obtained from [38], [47], [2], and [48] (see the breakdown of the known breast cancer driver genes in Supplementary section 2 in S1 Text). The result of the validation is shown in Fig 10. From Fig 10, in the cases of the top 50 and top 100 predicted cancer drivers, CBNA outperforms all the other three methods. In the cases of the top 150 and top 200 predicted cancer drivers, CBNA still outperforms DriverNet and DawnRank and has similar or slightly lower performance compared to NetSig.

### Ranking mutated coding drivers predicted by CBNA based on mutation density

Although CBNA outperforms other the existing methods in identifying mutated coding cancer drivers, it contains false positive drivers, whose mutation frequency is high, due to their length such as *TTN* and *DMD*. Thus, to eliminate these long genes out of the top hits, we have implemeted another option to rank predicted mutated cancer drivers based on mutation density (i.e. the ratio of mutation count and gene length). The top 20 mutated coding drivers using mutation density are listed in Table 1.

**Table 1:** The top 20 mutated coding drivers using mutation density.

No.	Predicted driver	Mutation density	In CGC?
1	*TP53*	0.0127275	✓
2	*PIK3CA*	0.0041422	✓
3	*GATA3*	0.0034876	✓
4	*HLA-C*	0.0026619	
5	*GPS2*	0.0025943	
6	*ICAM4*	0.0025526	
7	*UBC*	0.0024289	
8	*STUB1*	0.0022676	
9	*GPRASP2*	0.0019795	
10	*APOA1*	0.0018190	
11	*GPRASP1*	0.0013765	
12	*IRF3*	0.0012686	
13	*GDF9*	0.0012274	
14	*RPL18A*	0.0011784	
15	*GP1BA*	0.0010925	
16	*HLA-A*	0.0010813	✓
17	*HSPA8*	0.0010451	
18	*MED12*	0.0010043	✓
19	*EEF1A1*	0.0009942	
20	*KRT18*	0.0009913	

### Discovering coding cancer drivers without mutations and miRNA cancer drivers

**Fig 11:**
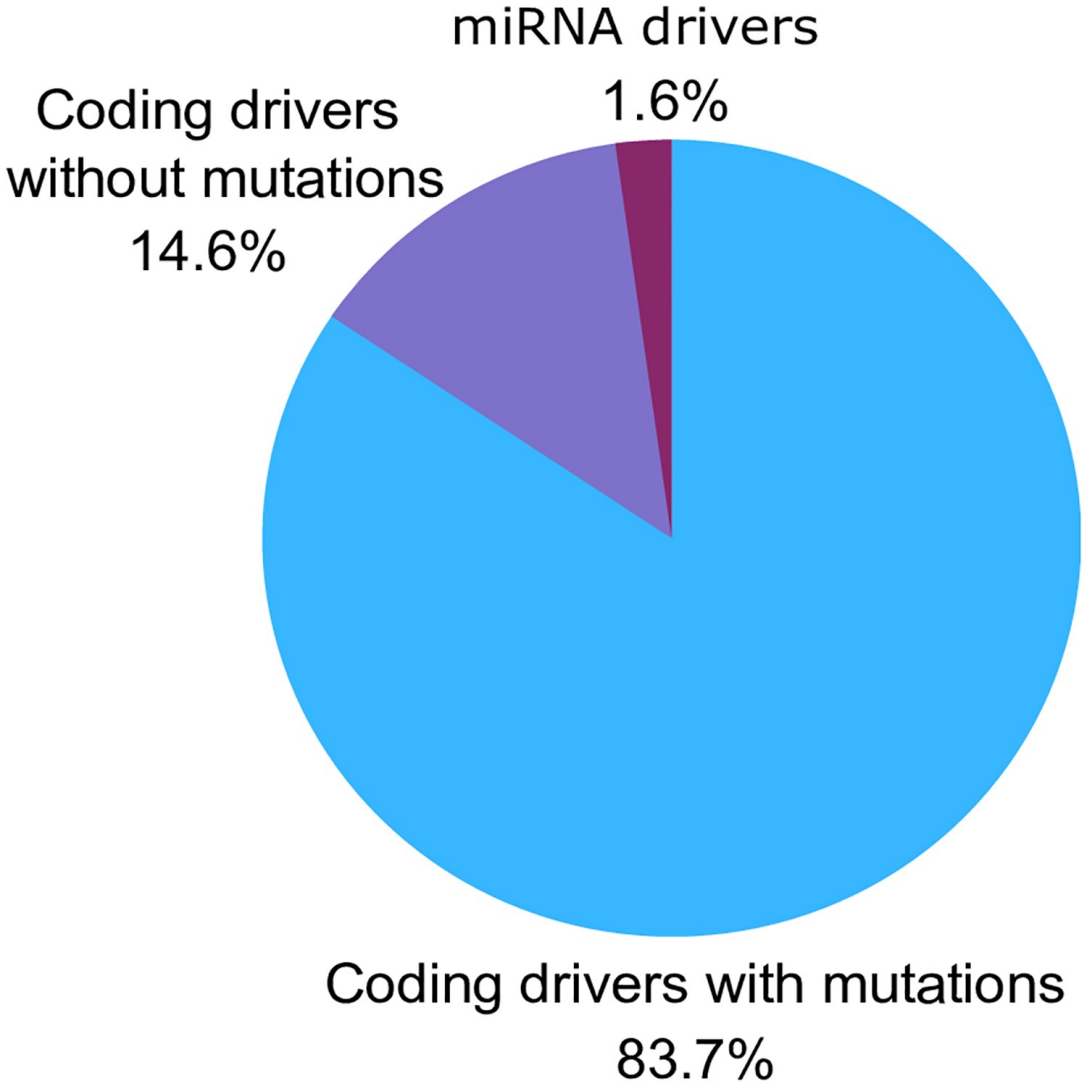
Identification of coding and miRNA cancer drivers. The chart shows the percentage of different types of cancer drivers identified by CBNA from the BRCA dataset.

The percentage of coding and miRNA cancer drivers uncovered by CBNA is presented in Fig 11. In addition to discovering coding cancer drivers with mutations, CBNA also has the ability to identify coding cancer drivers without mutations and miRNA cancer drivers (see the list of coding drivers without mutations in S2 Table and list of miRNA drivers in S3 Table). It can be seen from Fig 11 that only 1.6% of drivers predicted by CBNA are miRNA drivers. One of the reasons is that the number of coding genes in the network used by CBNA is much more than the number of miRNAs. In addition, as illustrated in Fig 2, candidate drivers play the central roles in the network rather than only regulating other nodes (i.e. a gene/node which has more incoming and outgoing edges in the network has higher chance to be a driver gene). However, miRNAs do not have high enough incoming edges as they regulate TFs and mRNAs, but only a small amount of miRNAs are regulated by TFs.

Most of the coding candidate drivers without mutations are novel drivers since the existing methods are not designed for finding drivers without mutations. As a result of lacking groundtruth, we use GO [49] enrichment analysis for the evaluation of CBNA in detecting coding cancer drivers without mutations (see the detail of the enrichment analysis in Supplementary section 3 in S1 Text). The enrichment analysis shows that several predicted drivers are significantly associated with enriched terms in GO biological process and GO molecular function, suggesting that the findings of our method are biologically meaningful. Among the top predicted coding drivers involved in GO biological process and molecular function, *CDC42* is the most promising potential cancer driver. There has been evidence that *CDC42* plays a critical role in promoting breast cancer cell invasion and forming invadopodia by activating N-WASp [50]. This gene is enriched in many GO terms, including nucleoside-triphosphatase activity, purine ribonucleoside binding, guanyl ribonucleotide binding, etc. Other drivers predicted by our method, including *IRS2* and *SUMO2*, have been shown by previous studies [51, 52] to be related to breast cancer.

CBNA also identifies 17 potential miRNA cancer drivers. Eleven of them have been confirmed as miRNAs related to tumorigenesis of BRCA by OncomiR [53], a resource for studying pan-cancer miRNA dysregulation. Especially, among these 11 miRNAs, *hsa-miR-342-5p* has been proved to be involved in progressing breast cancer in another work [54]. According to [54], *hsa-miR-342-5p* is a regulator of the growth of breast cancer cells. Besides, other miRNAs predicted by CBNA are also potential drivers, such as *hsa-miR-130a-5p* and *hsa-miR-223-5p*. *hsa-miR-130a-5p* targets *FOSL* and upregulates *ZO-1* in order to suppress breast cancer cell migration [55] and *hsa-miR-223-5p* is a coordinator of breast cancer [56]. The summary of predicted miRNA BRCA drivers is shown in Table 2.

**Table 2:** miRNA BRCA drivers predicted by CBNA.

No.	Predicted driver	Confirmed	References
1	*hsa-miR-130a-5p*	✓	[55]
2	*hsa-miR-141-5p*	✓	[53]
3	*hsa-miR-142-5p*	✓	[53]
4	*hsa-miR-181a-5p*	✓	[53]
5	*hsa-miR-214-5p*	✓	[53]
6	*hsa-miR-222-5p*		
7	*hsa-miR-223-5p*	✓	[56]
8	*hsa-miR-23a-5p*	✓	[53]
9	*hsa-miR-338-5p*	✓	[53]
10	*hsa-miR-342-5p*	✓	[53, 54]
11	*hsa-miR-3614-5p*	✓	[53]
12	*hsa-miR-3648*	✓	[53]
13	*hsa-miR-429*	✓	[53]
14	*hsa-miR-4473*		
15	*hsa-miR-4757-5p*		
16	*hsa-miR-663a*		
17	*hsa-miR-9-5p*	✓	[53]

### Identifying drivers in different conditions

Since the biological condition of cancer patients is different from that of healthy people, the important genes in cancer and non-cancer condition might be different. It means that some genes become critical in the cancer condition while they are of little importance in the normal condition. We concern these genes as they are specific for the tumour state. Hence, we systematically analyse driver genes which are particular for the cancer condition only. Due to the fact that CBNA can identify drivers for a network in a condition, we apply CBNA to detect regulators in the normal condition. Specifically, instead of using the expression data of breast cancer patients, we use the expression data of normal samples to build the network (see the detail of the network in Supplementary section 4 in S1 Text). The drivers identified by CBNA based on this network are regulatory genes in the normal condition. Then we compare them to the driver genes of the cancer condition to uncover cancer drivers which are only specific to the cancer state. As in this approach, there are few coding drivers without mutations and miRNA drivers are similar to those in the original approach, we only focus on coding drivers with mutations. We validate these predicted coding cancer drivers with mutations (i.e. only specific to cancer condition) against the CGC and there are 45 validated drivers (see the list of these 45 drivers in S4 Table).

### Exploring drivers for cancer subtypes

As breast cancer has several subtypes with different morphologies and clinical outcomes, the subtypes might have different causes and drivers. In this section, we explore candidate drivers for breast cancer subtypes using CBNA. Firstly, we categorise the 747 breast cancer samples into different subtypes using the Pam50 method [57, 58]. As a result, we have 221 samples in Luminal A subtype, 165 samples in Luminal B subtype, 158 samples in Basal subtype, 108 samples in Her2 subtype, and 95 samples in Normal-like subtype. Then, we apply CBNA to these subsets respectively in order to identify drivers for each subtype of breast cancer. The predicted drivers which are specific to only one subtype of breast cancer are listed in Table 3.

**Table 3:** Predicted drivers which are specific to each breast cancer subtype.

Subtype	Coding drivers with mutations (Top 10)	Coding drivers without mutations	miRNA drivers
Luminal A	*PTEN, RUNX1, MED12, ATM, DSP, SF3B1, ATXN2, CHD3, HTT, NCOA6*	*ADAM17, APEH, ARF6, AVP, AVPR1A, BARX1, BTBD2, CAMLG, CSF2, DLEU1, EXOSC6, F2RL3, GADD45A, HBEGF, HLA-DRA, IFNGR2, MMP7, PAFAH1B2, PPP1R9B, RNF216, RNF7, RPS27A, SOX4, STMN2, TGOLN2, TNFRSF13C, WASH2P*	*hsa-let-7i-5p, hsa-miR-130a-5p, hsa-miR-181a-5p, hsa-miR-196b-5p, hsa-miR-199b-5p, hsa-miR-24-1-5p, hsa-miR-548aw, hsa-miR-92a-1-5p, hsa-miR-99a-5p*
Luminal B	*GATA3, ERBB2, TAF1, HDAC6, SETDB1, DLG1, EP400, UBC, ARHGEF12, FN1*	*ARF3, DCP1A, IL2, POMC, RAB5A, RAB5B, RAP1GAP, RASSF8, SSSCA1, TNFSF12, UBE2E2, VBP1, YWHAH*	*hsa-let-7a-5p, hsa-miR-107, hsa-miR-128-1-5p, hsa-miR-142-5p, hsa-miR-148b-5p, hsa-miR-361-5p, hsa-miR-548s, hsa-miR-616-5p, hsa-miR-647, hsa-miR-766-5p, hsa-miR-93-5p, hsa-miR-939-5p*
Basal	*SYNE1, LRP2, LRP1, FLNA, RB1, GOLGA4, SPTAN1, ATN1, CBLB, CREBBP*	*GTF2B, MTA1, NR0B1, PBX2, RAB8A, RIT1, S100A8, SNTB2, TAF15, TCEB2, TXNDC17*	*hsa-miR-17-5p, hsa-miR-18a-5p, hsa-miR-20a-5p, hsa-miR-20b-5p, hsa-miR-30c-5p, hsa-miR-3646, hsa-miR-425-5p, hsa-miR-4778-5p, hsa-miR-6759-5p*
Her2	*DMD, AKAP9, PRKDC, FBN1, BRCA2, PIK3R1, HSPG2, PTPN13, TLN1, NUP98*	*AKAP5, BIRC5, CLTB, EIF3J, GFRA1, HLA-DPA1, MAFK, NKX2-1, PDE6G, RBX1, RRAS2, SET, TBPL1, TERF1*	*hsa-miR-146a-5p, hsa-miR-148a-5p, hsa-miR-150-5p, hsa-miR-181b-5p, hsa-miR-24-2-5p, hsa-miR-25-5p, hsa-miR-326, hsa-miR-5698, hsa-miR-6783-5p, hsa-miR-7-5p, hsa-miR-9-5p*
Normal-like	*HIPK2, SMARCC1, COL1A1, PPP3CA, GOLGA2, ETS1, KPNB1, TUBB2B, AKT1, C8orf33*	*CHMP4B, GRAP, GZMB, PIM1, PLA2G10, POLDIP2, PSMC1, PSME3, PTK6, RAC3, RPSA, SCT, SIGIRR, VASP, WNT1*	*hsa-miR-1307-5p, hsa-miR-141-5p, hsa-miR-29c-5p, hsa-miR-3605-5p, hsa-miR-6845-5p*

To obtain the list of predicted drivers, we use the subtype-specific mutations to rank the candidate drivers and for each subtype, only those candidate drivers which have dominant mutations in that subtype are included in the list of predicted drivers. Mutations of a gene are dominant in a subtype if they belongs to that subtype more than to all other subtypes. It has been shown that some promising subtype-specific cancer drivers predicted by CBNA are *PTEN* (Luminal A) and *FN1* (Luminal B). Luminal A tumours have a strong and diffuse expression of *PTEN* [59] and *FN1* plays pivotal roles in the tumorigenesis of Luminal B breast cancer by influencing the pathways in cancer [60].

### Detecting drivers of epithelial-mesenchymal transition

Metastasis, a migration process of cancer cells from the primary tumour, mainly causes the death of cancer patients. One process which creates these metastatic cells is epithelial-mesenchymal transition (EMT) [61]. There is evidence that EMT is promoted by coding RNAs [62] and/or non-coding RNAs [63]. Thus, in this section, we apply our proposed method to the BRCA dataset to detect drivers for breast cancer metastasis. Since our method identifies driver genes which control the mesenchymal condition, the identified driver genes are expected to drive the transition from epithelial state to mesenchymal state in breast cancer patients.

We firstly classify the 747 breast cancer samples into different phenotypes by using EMT score [41]. As a result, we have 189 epithelial samples, 461 mesenchymal samples, 44 intermediately epithelial samples, and 53 intermediately mesenchymal samples. We then apply CBNA to the 461 mesenchymal samples to build the network for the mesenchymal condition and discover drivers which cause this mesenchymal condition. Say in other words, these predicted drivers, called EMT drivers, regulate the transition from epithelial to mesenchymal.

We validate the top 100 predicted coding EMT drivers against mesenchymal genes in EMT signatures [41] and 17 predicted miRNA EMT drivers against pro-mesenchymal miRNAs in EMT miRNAs [42]. There are 7 validated coding and 6 validated miRNA drivers. The p-values of these overlaps between the predicted drivers and EMT genes/EMT miRNAs are significant at 0.007 and 1.333e-07 respectively (see the detail of epithelial-mesenchymal transition drivers in Supplementary section 5 in S1 Text).

There are several potential EMT drivers which are predicted by our method, such as *FYN*, *E2F1*, and *EP300*. *FYN* promotes mesenchymal phenotypes through STAT5/NOTCH2 signalling node in Basal breast cancer cells [62]. *E2F1* drives epithelial-mesenchymal transition by regulating *TXNIP* [64]. The downregulation of *EP300* is related to the initiation of an EMT [65]. The top 20 coding drivers and 17 miRNA drivers for EMT in breast cancer is shown in Table 4.

**Table 4:** Top 20 coding and 17 miRNA drivers predicted for EMT in breast cancer.

Coding drivers	miRNA drivers
*E2F1, FLI1, CREBBP, GATA1, ETS1, EZH2, E2F6, YWHAG, FOXO1, MBD3, CREB1, TCF3, EBF1, EP300, YWHAZ, MAZ, FYN, TAF1, SPI1, ATXN1*	*hsa-miR-128-2-5p, hsa-miR-130a-5p, hsa-miR-141-5p, hsa-miR-146a-5p, hsa-miR-181a-5p, hsa-miR-18a-5p, hsa-miR-223-5p, hsa-miR-23a-5p, hsa-miR-31-5p, hsa-miR-3614-5p, hsa-miR-3648, hsa-miR-4745-5p, hsa-miR-584-5p, hsa-miR-615-5p, hsa-miR-624-5p, hsa-miR-663a, hsa-miR-9-5p*

### Conclusion

Since cancer initialisation and progression are driven by not only coding drivers but also non-coding drivers, it urgently requires novel and effective methods to discover both coding and non-coding drivers to elucidate their regulatory mechanism for the development of powerful cancer treatments. With the fast development of computer science and DNA sequencing techniques, there are multiple computational methods developed to discover cancer drivers. However, most of the current methods detect coding cancer drivers with mutations while some genes, which do not contain mutations, regulate driver mutations to develop cancer, and some non-coding RNAs regulate gene expression and drive cancer.

To overcome the current limitations, we propose the novel method CBNA to discover cancer drivers based on the gene network information. The aim of CBNA is to integrate various types of genomic data such as gene expression, network information, and mutations to uncover both coding and non-coding drivers (i.e. miRNA drivers). Firstly, based on the gene expression of cancer patients and the existing databases of gene interactions, we build a network for a condition (i.e. the network for cancer condition). Then, we detect the minimum node subset which controls the whole network and the ultimate aim is to find critical nodes of the network. The critical nodes are nodes whose absence increases the size of the minimum node subset controlling the whole network. Because without the critical nodes, we need to control more nodes to control the whole network, the critical nodes play the central role of the network and they are likely candidate drivers for the condition of the network (i.e. cancer condition).

We have applied CBNA to the BRCA dataset to discover breast cancer drivers. Comparing to the existing methods, our method is more effective in uncovering coding cancer drivers with mutations as validated by using the CGC. Our method can also identify coding cancer drivers without mutations as well as miRNA cancer drivers. In addition, the proposed method can be applied to explore drivers for cancer subtypes and drivers for epithelial to mesenchymal transition. Take together, we believe the proposed method is a complement for the existing methods in identifying cancer drivers and it can provide new insights of the molecular regulatory mechanisms of cancer initialisation and progression. Thus, it has the potential to contribute significantly to the design of effective treatment strategies for cancer patients.

## Supporting information

S1 Table List of coding drivers with mutations. Top 200 predicted coding drivers with mutations in breast cancer by CBNA.
(CSV)

S2 Table List of coding drivers without mutations. Coding BRCA drivers without mutations predicted by CBNA.
(CSV)

S3 Table List of miRNA drivers. Predicted miRNA BRCA drivers by CBNA.
(CSV)

S4 Table List of validated coding drivers with mutations. Validated coding BRCA drivers with mutations which are specific to the cancer condition only.
(CSV)

S1 Text Supplementary information. (PDF)
