# Supplementary material for: CBNA: A control theory based method for identifying coding and non-coding cancer drivers
Source: PLoS Comput Biol. 2019 Dec 2;15(12):e1007538. doi: 10.1371/journal.pcbi.1007538 (PMC6907873; doi:10.1371/journal.pcbi.1007538)
Supplement: S1 Text — (PDF) [file pcbi.1007538.s005.pdf]

Supplementary information of CBNA: A control theory based method for identifying coding and non-coding cancer drivers

Vu VH Pham[1], Lin Liu[1], Cameron P Bracken[2,3], Gregory J Goodall[2,3], Qi Long[4], Jiuyong Li[1*], Thuc D Le[1*]

1 School of Information Technology and Mathematical Sciences, University of South Australia, Mawson Lakes, SA 5095, Australia
2 Centre for Cancer Biology, an alliance of SA Pathology and University of South Australia, Adelaide, SA 5000, Australia
3 Department of Medicine, The University of Adelaide, Adelaide, SA 5005, Australia
4 Perelman School of Medicine, University of Pennsylvania, Philadelphia, PA 19104, USA

* Jiuyong.Li@unisa.edu.au or Thuc.Le@unisa.edu.au

1 Using different methods for ranking predicted coding cancer drivers based on mutations

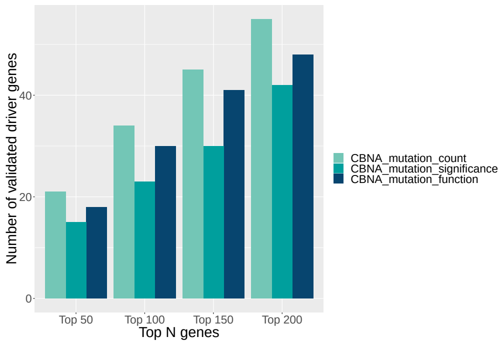

Fig A. CBNA with different methods for ranking predicted coding cancer drivers based on mutation burden The coding cancer drivers predicted by CBNA are ranked based on (1) mutation count, (2) mutation significance of mutation frequency and spectrum of patients, and (3) functional impact of mutations. The results of the methods are validated against the CGC. Each bar shows the number of validated cancer drivers of each method.

The first approach to rank candidate coding cancer drivers is based on mutation count. The second approach is based on (i) mutation frequency and spectrum of patients, and (ii) mutation rates of genes incorporating expression level and replication time, as the MutSigCV method does [1]. Another approach to rank cancer drivers predicted by CBNA is based on the functional impact of mutations as OncodriveFM does [2]. We compare these approaches and the result is illustrated in Fig. A. It can be seen that the performance of CBNA using mutation count is better than the other methods in all the cases.

2 Breakdown of known breast cancer driver genes

There are 80 known breast cancer genes in the well-curated set which are obtained from [3], [4], [5], and [6]. Of these 80 genes, 18 are filtered out by the PPI and miRNA databases. For the remaining gene set, 19 genes are critical nodes, 8 are redundant nodes, and 35 are ordinary nodes in the network created by CBNA.

3 Several genes enriched significantly in biological processes and molecular functions

We conduct GO [7] enrichment analysis of coding non-mutation drivers for breast cancer, which are predicted by our method. There are several predicted drivers which are involved in various GO biological processes and GO molecular functions. We rank enriched terms of GO biological process as well as GO molecular function based on the number of the predicted drivers in an enriched term. The higher number of the predicted drivers an enriched term has, the higher it is in the list. Then we select the top 10 GO process terms and top 10 GO function terms. Among genes in the top GO processes or top GO functions, we rank genes based on their occurrence in these enriched terms. The higher number of occurrences a gene has, the higher it is in the list.

The heatmap of enrichment profiles for the top 20 predicted coding drivers of the top 10 GO process terms and top 10 GO function terms is shown in Fig. B. The significant enrichment of predicted coding non-mutation drivers predicted by CBNA suggests that these drivers are important in various biological processes and molecular functions. Say in other words, the findings of our method are biologically meaningful.

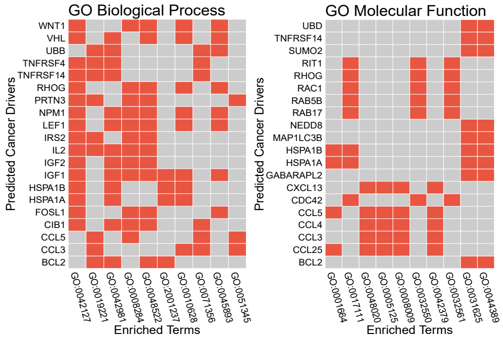

Enriched Terms	Biological Process
GO:0042127	regulation of cell proliferation
GO:0019221	cytokine-mediated signaling pathway
GO:0042981	regulation of apoptotic process
GO:0008284	positive regulation of cell proliferation
GO:0048522	positive regulation of cellular process
GO:2001237	negative regulation of extrinsic apoptotic signaling pathway
GO:0010628	positive regulation of gene expression
GO:0071356	cellular response to tumor necrosis factor
GO:0045893	positive regulation of transcription, DNA-templated
GO:0051345	positive regulation of hydrolase activity

Enriched Terms	Molecular Function
GO:0001664	G-protein coupled receptor binding
GO:0017111	nucleoside-triphosphatase activity
GO:0048020	CCR chemokine receptor binding
GO:0005125	cytokine activity
GO:0008009	chemokine activity
GO:0032550	purine ribonucleoside binding
GO:0042379	chemokine receptor binding
GO:0032561	guanyl ribonucleotide binding
GO:0031625	ubiquitin protein ligase binding
GO:0044389	ubiquitin-like protein ligase binding

Fig B. Enrichment profiles of predicted coding cancer drivers The figure shows the top 20 predicted coding cancer drivers of the top 10 enriched terms in the context of GO biological process and GO molecular function. Red cells in the matrix illustrate that a driver is related to an enriched term (i.e. in a GO biological process or in a GO molecular function).

4 Characterising the controllability of the miRNA-TF-mRNA network for the normal state

The miRNA-TF-mRNA network of the normal state obtained by CBNA consists of 7,726 nodes and 31,053 directed edges. We apply the network control [8] to evaluate the controllability of the network by identifying its MDNS. We find that the size of MDNS, denoted as N_D, is 2,680, accounting for 45.5% of nodes. We then classify nodes as critical, ordinary, and redundant based on the change of N_D upon their removal. In the miRNA-TF-mRNA network of normal state, 11.2% of nodes are critical, 34% are ordinary, and the remaining 54.8% are redundant (Fig. C(A)). We find that critical nodes have higher in-degrees/out-degrees compared with ordinary and redundant nodes, which can be seen in the average in-degree/out-degree and accumulative in-degree/out-degree distribution of nodes in Fig. C(B) and Fig. C(C).

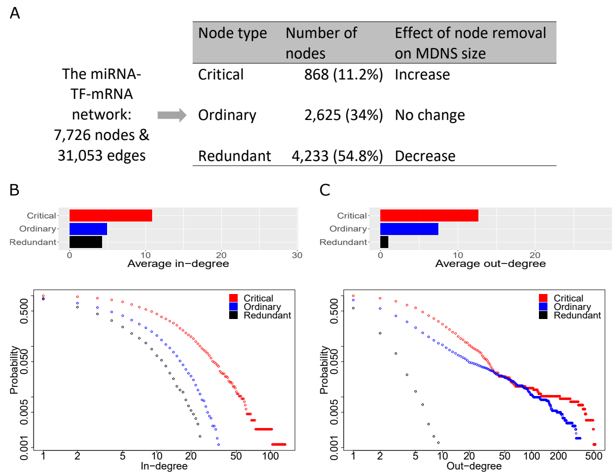

Fig C. Characterising the controllability of the miRNA-TF-mRNA network of normal state (A) Identification of critical, ordinary, and redundant nodes in the network. (B) Average in-degree and *accumulative* in-degree distribution for three different node types. (C) Average out-degree and *accumulative* out-degree distribution for three different node types.

5 Epithelial-mesenchymal transition drivers

We apply CBNA to BRCA dataset to identify epithelial-mesenchymal transition (EMT) drivers. These drivers are expected to drive the transition from epithelial state to mesenchymal state in breast cancer patients.

To validate the result, we rank predicted EMT drivers based on their node degree (i.e. the number of edges of a node) in the network of the mesenchymal condition, which is built by our method. The higher degree a driver has, the higher it is in the ranked driver list. We then validate the top 100 predicted coding EMT drivers with mesenchymal genes in EMT signatures [9] and 17 predicted miRNA EMT drivers with pro-mesenchymal miRNAs in EMT miRNAs [10]. There are 7 predicted coding EMT drivers and 6 predicted miRNA EMT drivers in these known EMT gene lists. The p-values are significant at 0.007 and 1.333e-07 respectively based on the hypergeometric test. The p-value is calculated as the following.

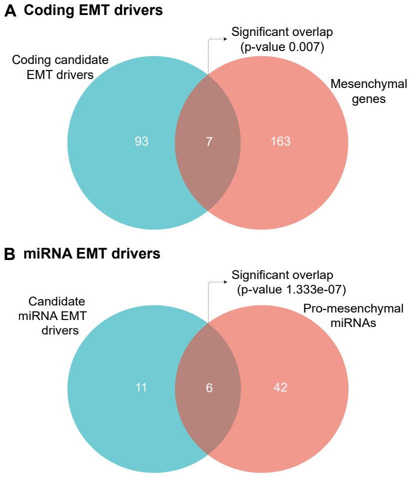

Fig D. Overlaps between candidate EMT drivers and known EMT genes The chart shows the significant ovelaps of (A) coding candidate EMT drivers (top 100) and mesenchymal genes, (B) candidate miRNA EMT drivers and pro-mesenchymal miRNAs.

$$p = 1 - \sum_{x=0}^{n-1} \frac{\binom{K}{x}\binom{N-K}{M-x}}{\binom{N}{M}}, \tag{1}$$

where N denotes the number of all genes of interest, K is the number of confirmed drivers (i.e. genes in known EMT gene lists), M is the number of estimated drivers, and n is the number of drivers validated.

The result is illustrated in Fig. D.

References

1. Lawrence MS, Stojanov P, Polak P, Kryukov GV, Cibulskis K, Sivachenko A, et al. Mutational heterogeneity in cancer and the search for new cancer-associated genes. Nature. 2013;499(7457):214–218. doi:10.1038/nature12213.

2. Gonzalez-Perez A, Lopez-Bigas N. Functional impact bias reveals cancer drivers. Nucleic Acids Research. 2012;40(21):e169–e169. doi:10.1093/nar/gks743.

3. Futreal PA, Coin L, Marshall M, Down T, Hubbard T, Wooster R, et al. A CENSUS OF HUMAN CANCER GENES. Nature reviews Cancer. 2004;4(3):177–183. doi:10.1038/nrc1299.

4. Cancer Genome Atlas N. Comprehensive molecular portraits of human breast tumours. Nature. 2012;490(7418):61–70. doi:10.1038/nature11412.

5. Stephens PJ, Tarpey PS, Davies H, Van Loo P, Greenman C, Wedge DC, et al. The landscape of cancer genes and mutational processes in breast cancer. Nature. 2012;486:400.

6. Pereira B, Chin SF, Rueda OM, Vollan HKM, Provenzano E, Bardwell HA, et al. The somatic mutation profiles of 2,433 breast cancers refines their genomic and

transcriptomic landscapes. Nature communications. 2016;7:11479–11479. doi:10.1038/ncomms11479.

7. Harris MA, Clark J, Ireland A, Lomax J, Ashburner M, Foulger R, et al. The Gene Ontology (GO) database and informatics resource. Nucleic Acids Res. 2004;32(Database issue):D258–61. doi:10.1093/nar/gkh036.

8. Liu YY, Slotine JJ, Barabási AL. Controllability of complex networks. Nature. 2011;473:167.

9. Tan TZ, Miow QH, Miki Y, Noda T, Mori S, Huang RY, et al. Epithelial-mesenchymal transition spectrum quantification and its efficacy in deciphering survival and drug responses of cancer patients. EMBO Mol Med. 2014;6(10):1279–93. doi:10.15252/emmm.201404208.

10. Cursons J, Pillman KA, Scheer KG, Gregory PA, Foroutan M, Hediyeh-Zadeh S, et al. Combinatorial Targeting by MicroRNAs Co-ordinates Post-transcriptional Control of EMT. Cell Systems. 2018;7(1):77–91.e7. doi:https://doi.org/10.1016/j.cels.2018.05.019.
